# Design and Locomotion Study of Stick-Slip Piezoelectric Actuator Using Two-Stage Flexible Hinge Structure

**DOI:** 10.3390/mi12020154

**Published:** 2021-02-04

**Authors:** Zheng Li, Zhirong Su, Liang Zhao, Haitao Han, Zhanyu Guo, Yuyang Zhao, Hexu Sun

**Affiliations:** Department of Electrical Engineering, School of Electrical Engineering, Hebei University of Science and Technology, Shijiazhuang 050018, China; suzhirong@stu.hebust.edu.cn (Z.S.); zhaoliang@stu.hebust.edu.cn (L.Z.); hanhaitao@stu.hebust.edu.cn (H.H.); guozhanyu@stu.hebust.edu.cn (Z.G.); zhaoyuyang@hebust.edu.cn (Y.Z.)

**Keywords:** piezoelectric actuator, stick-slip type, displacement amplification structure, clamping force

## Abstract

A novel piezoelectric actuator using a two-stage flexure hinge structure is proposed in this paper, which is used in a compact and high-precision electromechanical field. The two-stage flexure hinge structure is used to provide horizontal thrust and vertical clamping force to the driving feet, which solves the problems of unstable clamping force and insufficient load capacity in traditional stick-slip piezoelectric actuators. Firstly, the main structure of the driver and the working process under the triangular wave excitation voltage are briefly introduced. Secondly, after many simulation tests, the structure of the actuator is optimized and the stability of the structure in providing clamping force is verified. Finally, through the research of the operating performance, when the amplitude is 150 V and the frequency is 3.25 kHz as the excitation source, the maximum speed can reach 338 mm/s and can bear about 3 kg load. It can be seen from the analysis that the two-stage flexure hinge structure can improve the displacement trajectory.

## 1. Introduction

Piezoelectric actuators (PEAs) have the merits of strong electromagnetic compatibility, small size, high control precision, large output force, flexible design, and self-locking, etc. [1,2,3,4]. They are often widely used in precision manufacturing [5,6,7], aerospace, biomedical engineering, and other fields [8,9,10]. Since the output displacement of a piezoelectric device is small relative to the size of the device itself, most piezoelectric actuators are designed with the concept of gradually accumulating small displacements to achieve large displacements. PEAs can be separated into direct drive type, inchworm type, resonant type, etc. [11,12,13,14,15]. According to its working principle, each type of PEA has its relative merits and disadvantages. The direct-drive type has a high positioning resolution. Nevertheless, its output performance is limited [16]. The high-speed motion performance of the resonant type is superior, but the problems of wear and heat are still to be solved [17,18]. Compared with the inchworm type, the structure of the stick-slip type is relatively simple and easy to control, but its output performance is weak [19,20].

In order to deal with the shortcomings of instable clamping force and insufficient load capacity, a novel PEA using a two-stage flexure hinge structure is proposed in this paper. The PEA increases the telescopic moving distance through its leverage magnification structure. Then, the triangular flexure hinge structure is used to generate bidirectional coupling displacement to drive the slider to move. By testing the angle of flexure hinge structure, the optimal solution is obtained. By referring to the experimental part of the relevant literatures [21,22,23,24,25,26], the experimental platform for measuring the performance data of the PEA is constructed. The PEA will greatly increase the speed and displacement of the driven object. 

The following is a brief description of the article. Firstly, the composition and operating principle of the PEA are briefly introduced. Secondly, the derivation and calculation of the theoretical formula are carried out first, and then the simulation verification shows that the PEA has the merit of improving the clamping force. The structure of the lever in the PEA is parameterized to improve the output performance of the PEA. Thirdly, the operation performance of the PEA is studied to find and analyze its optimal operation state. Finally, the article is summarized.

## 2. Composition and Operating Principle

### 2.1. Composition

**Figure 1:**
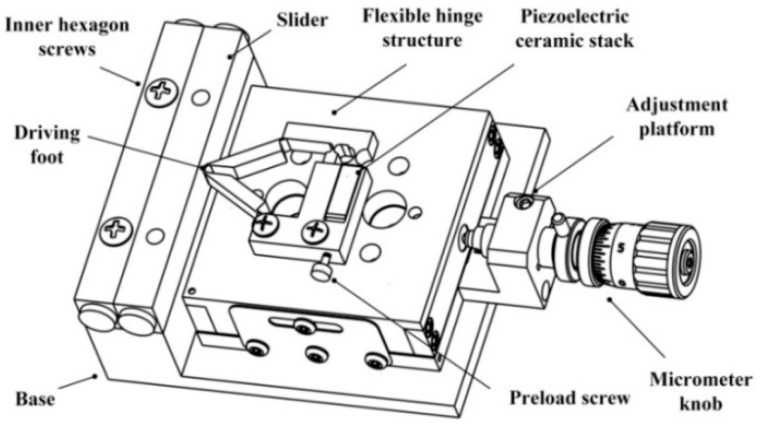
Structural model of the piezoelectric drive platform.

The piezoelectric driving platform is composed of a piezoelectric ceramic stack, a cross-roller linear guide, a adjustment platform, a flexure hinge structure, and a pedestal. Figure 1 illustrates the structure of each part. The cross-roller linear guide is fixed on the adjustment platform with screws. The piezoelectric stack is closely connected with the flexure hinge structure, and the preload is adjusted by fastening screws. Use the micrometer knob to modify the position of the cross-roller linear guide to change the pre-tightening force between them.

**Figure 2:**
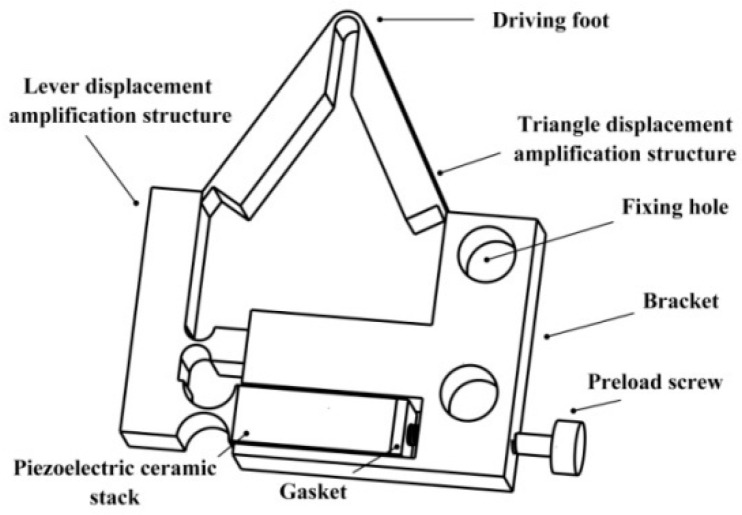
Structural model of the driving part.

Figure 2 shows the prototype of the proposed actuator. It consists of a bracket, a two-stage flexible hinge structure, a piezoelectric ceramic, a gasket, and a pre-tightening screw. The gasket is added between the pre-tightening screw and the piezoelectric ceramic. Prevent the pre-tightening screw directly acting on the piezoelectric ceramic stack. Two fixing holes on the bracket fix the piezoelectric driving component to the platform.

**Figure 3:**
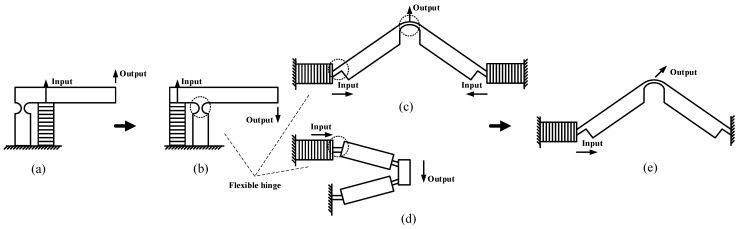
Schematic diagram of micro displacement amplification structure: (**a**) Lever displacement amplification structure; (**b**) Changed lever displacement amplification structure; (**c**) Triangle displacement amplification structure; (**d**) Four-bar displacement amplification structure; (**e**) Changed triangle displacement amplification structure.

The two-stage flexible hinge structure is composed of the lever displacement amplification structure and triangle displacement amplification structure. The amplification structure uses the characteristics of flexible hinge and the physical principle of the structure to increase the output displacement. Flexible hinge is an integrated structure with an arc or rectangular notch, as shown in the dotted circle in Figure 3. Flexible hinge has the characteristics of rapid recovery of elastic deformation, small volume, no mechanical friction, and high sensitivity, so it is often used in the movement of small angular displacement around the axis. The amplification structure makes use of this characteristic of flexible hinge [27]. 

The common amplification structures are the lever displacement amplification structure, triangle displacement amplification structure(bridge displacement amplification structure), and four-bar displacement amplification structure. Figure 3a shows the common lever displacement amplification structure, which has the characteristics of simple structure, few flexure hinges, and high amplification efficiency. The magnification can be changed by changing the position of the piezoelectric stack laterally. In order to adapt to the structure of the actuator and reduce the volume of the actuator, the structure in Figure 3a is adjusted appropriately, and the changed structure is shown in Figure 3b.

Figure 3c shows the triangle amplification structure, which can enlarge the displacement in the vertical direction. Changing the angle of the triangle can change the magnification. The smaller the angle, the larger the magnification. However, the smaller the inclination angle, the greater the stress of the hinge, and the service life will be affected.

Figure 3d is a four-bar amplification structure. Its structure is different from that of the triangle structure, which is driven by two piezoelectric stacks. Only one piezoelectric stack is retained and the direction of its displacement output is changed. The hinge on the side of the cancelled piezoelectric stack is fixed, and the number of hinges has increased. Compared with the structure in Figure 3c, this structure changes the output direction. Combined with the characteristics of the structures in Figure 3c,d, the structure shown in Figure 3e is improved to realize the stable output of bidirectional displacement.

### 2.2. Operating Principle

**Figure 4:**
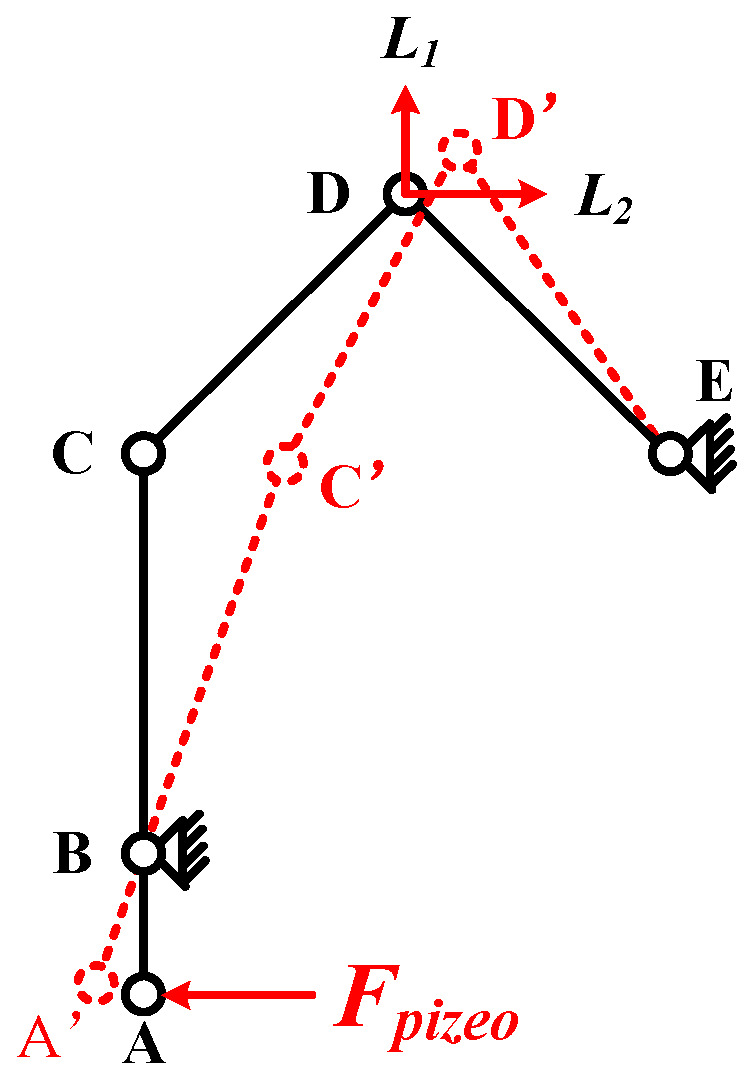
Schematic diagram of the movement of the driving part.

The diagrammatic sketch in Figure 4 shows the deformation of piezoelectric ceramic stack acts on point A in the process of stretching. Point C moves to point C′ by lever amplification. The generated pressure deforms the triangular flexible hinge structure, so that the driving point D moves to D′. The displacement *L*_1_ of the driving point D provides the preload for the slider, and the displacement *L*_2_ causes the slider to move laterally.

The PEA adopts the two-stage flexible hinge structure, in which the characteristics of piezoelectric ceramics are fully utilized. The amplified displacement produces the lateral and longitudinal coupling displacement through the triangular flexible hinge structure, which drives the slider to move laterally. The deformation of the piezoelectric ceramic stack, flexible hinge, and slider is at the micron level. The operating principle of the PEA can be better described by amplifying the displacement produced at each place.

**Figure 5:**
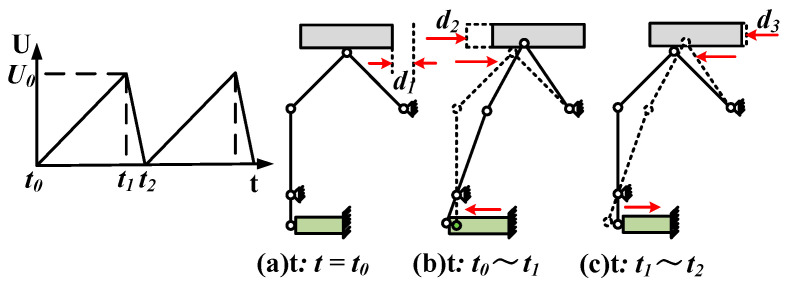
Piezoelectric actuator workflow: (**a**) initial state; (**b**) progressive elongation of the piezoelectric ceramic stack; (**c**) rapid shrinkage of the piezoelectric ceramic stack.

A positive driving voltage with oblique waveform is exerted on the piezoelectric stack, and the symmetry of the waveform is 90%, as shown in Figure 5. In a cycle, the initial and final values of voltage are 0 V, *U*_0_ is the peak to peak value of voltage. Make sure the positive voltage is exerted on the piezoelectric stack to produce tensile deformation. According to the characteristics of piezoelectric stack, the larger the *U*_0_ value, the larger the displacement due to tension.

Figure 5a shows the initial moment is *t*_0_. The adjusting platform provides the required preload on the contact surface of the linear guide. From *t*_0_ to *t*_1_, the piezoelectric stack is in a state of gradual tension. The flexible hinge structure is deformed due to the push of the piezoelectric ceramic stack. The slider moves to the right driven by friction, resulting in displacement *d*_2_, as shown in Figure 5b.

In the interval from *t*_1_ to *t*_2_, the piezoelectric ceramic stack is in a state of rapid contraction. The structure of the flexible hinge recovered quickly. The extrusion force on the contact surface of the linear guide is weakened. The driving foot had left friction on the slider. Due to the inertial action, the slider has a small displacement *d*_3_ or no displacement to the left, as shown in Figure 5c. In a period, the displacement of the slider moving to the right is *d*_1_ (*d*_1_ = *d*_2_ − *d*_3_), and the slider moves in step linear motion.

### 2.3. Force Analysis

**Figure 6:**
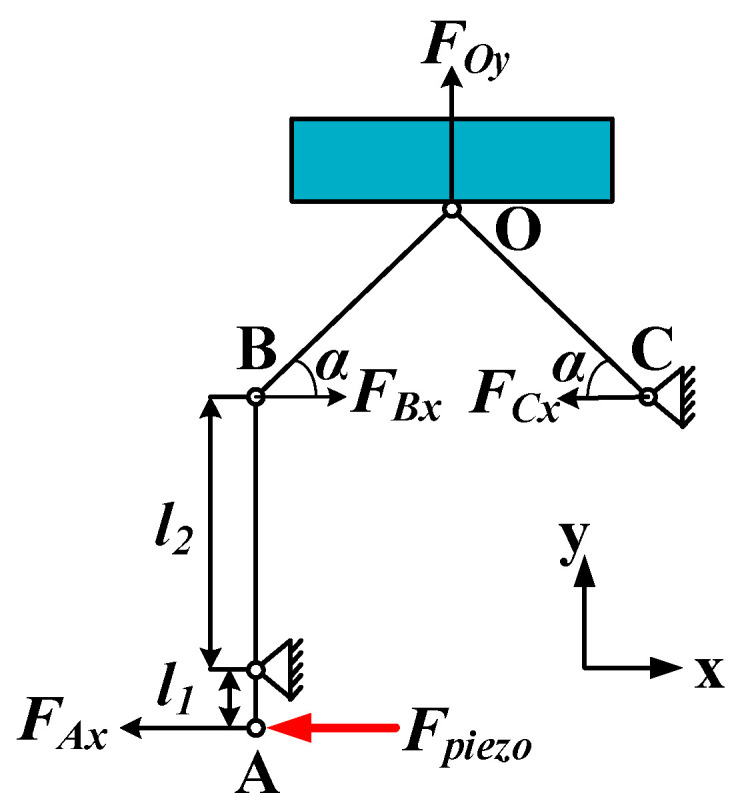
Force analysis of the flexible hinge structure.

For proving the merits of the PEA in theory, to create the flexible hinge structure model of the PEA, the force during the driving process was analyzed, as shown in Figure 6. The rectangle at the top of the graph represents the mover, the x-axis represents the direction of the mover’s movement, and the y-axis is the direction perpendicular to the motion direction.

According to the conservation of moment in the lever principle, the lateral displacement is amplified under the action of the lever. The amplification factor *k* can be expressed by the following equation [28]:

(1) $$k=\frac{l_{2}}{l_{1}}$$

In Formula (1), *l*_1_ indicates the distance from point A to the fulcrum, and *l*_2_ indicates the distance from point B to the fulcrum, as shown in Figure 6. With other parameters unchanged, reducing *l*_1_, or increasing *l*_2_ will increase the magnification, but at the same time, it will also have greater requirements on the stiffness of lever material.

The flexible hinge structure transforms the horizontal displacement at point A into the horizontal displacement and longitudinal displacement at point O by hinge rotation. Assuming that the translational and rotational joints in the flexible hinge structure are in an ideal state, according to the force analysis, it can be concluded that:

(2) $$F_{Ax}=F_{piezo},$$

(3) $$F_{Bx}=\frac{F_{Ax}}{k},$$

(4) $$F_{Cx}=-F_{Bx},$$

(5) $$F_{Oy}=F_{Bx}\tan\alpha -F_{Cx}\tan\alpha =\frac{2}{k}F_{piezo}\tan\alpha .$$

**Figure 7:**
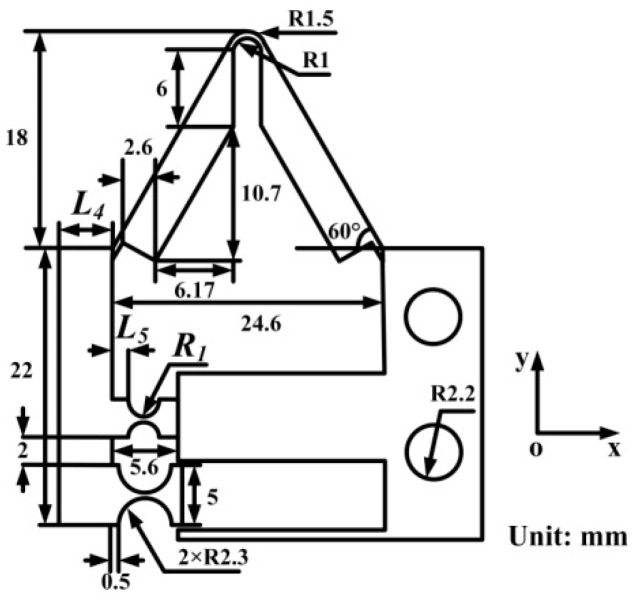
Main structural parameters of the flexible hinge mechanism.

According to Formula (4), the clamping force of the contact surface is related to the inclination α. By changing α, the clamping force can be changed, thus, enhancing the driving performance. According to the main parameters of the flexible hinge structure, the magnification is 2.1, as shown in Figure 7. The definition of the coordinate axis in the figure is consistent with that in Figure 6. When the extrusion force is equal to the output force produced by piezoelectric ceramic, the α can be calculated as 48.39° according to Formula (5). Therefore, when α ≥ 48.39°, F_Oy_ ≥ F_pizeo_.

## 3. Simulation Analysis

Finite element analysis (FEA) is the use of mathematical approximation principles to simulate physical systems, to achieve the purpose of solving problems by simplifying complex physical models. In this article, the FEA of the proposed flexible hinge structure is carried out by using ANSYS software. The established model is transformed into a finite element model and simulated. It is further verified that the hinge structure proposed in the theory can magnify the clamping force and horizontal displacement. The model is comprised of a cross-roller linear guide, a bracket, and a flexible hinge structure. Select fabricated steel for load-bearing as the material of the cross-roller linear guide; spring steel has the characteristics of high strength, high toughness, and high plasticity. The lever structure and the bracket are made of 65Mn; the aluminum alloy Al7075 with better strength and wear resistance is selected as the material of the triangular flexible hinge part. Table 1 shows the specific parameters of materials.

After the model is built, due to the same material, the bracket and the lever structure are integrated. The material of the triangle hinge structure is different from the connected structure, so it needs to be bonded. In order to approach the slight friction caused by relative motion in a real situation, the friction coefficient of the cross roller linear guide is set to 0.01. So, as to eliminate the normal relative displacement between contact surfaces, the behavior of the contact surface is selected as “no separation”; the friction coefficient between the flexible hinge structure and the slider is set to 0.2. The behavior of the contact surface is selected as “standard”.

Apply a force on the surface where the lever structure is connected to the piezoelectric stack. This pressure is used as the output of the piezoelectric ceramic stack. In order to approach the real output, the force value is set to 1000 N.

Optimize the lever magnification structure. The parameters in the structure are shown in the Figure 6. The initial setting hinge radius is 1.2 mm, the distance between the hinge and the lever connection is 2 mm. The static analysis of the flexure hinge structure with different lever width is carried out. With the increase of the lever width, the clamping force of the contact surface reaches the maximum at the lever width of 4.5 mm. The horizontal displacement of the slider changes with the clamping force, as shown in Table 2. When the lever width is insufficient, it is easy to bend and deform. If the width is too large, the loss of force will be increased. Therefore, if the lever width is too large or too small, it will hinder the transmission of force. The optimum lever width is 4.5 mm.

The initial setting lever width is 4.5 mm, hinge radius is 1.2 mm. The static analysis of flexure hinge structure with different hinge and lever connection distance is carried out. With the decrease of the distance between the hinge and the lever, the clamping force of the contact surface reaches the maximum at the horizontal displacement at 0 mm of the lever side, and increases with the horizontal displacement of the slider, as shown in Table 3. The closer the hinge position is to the lever, the higher the driving capacity of the piezoelectric actuator. The optimum distance between the hinge and the lever is 0 mm.

The initial setting lever width is 4.5 mm, the distance between the hinge and the lever connection is 0 mm. The static analysis of the flexure hinge structure with different hinge radius is carried out. As the radius of the hinge increases, the clamping force reaches the maximum when the radius is 1.4 mm. At the same time, the horizontal displacement of the slider increases, as shown in Table 4. The larger the hinge radius is in the allowable range, the smaller the loss of the lever structure on the hinge, which increases the horizontal displacement of the slider and improves the driving ability of the PEA. The optimum hinge radius is 1.4 mm.

In view of the above analysis, the optimal structure of the leverage amplification structure is finally obtained as the lever width is 4.5 mm, the hinge radius is 1.4 mm, the distance between the hinge and the lever connection is 0 mm. The contact surface clamping force can reach 1201 N, and the slider horizontal displacement is 142 μm.

The simulation results of ANSYS show the clamping force of 1201 N is higher than the set output of 1000 N. The results verify the superiority of the proposed flexure hinge structure. However, the clamping force calculated by Formula (4) is 1540 N. For the clamping force, the simulation result is less than the theoretical calculation result. There are two main reasons for the force loss. One is the deformation caused by the linear or rotary motion of the flexure hinge; the other is the slight deformation of the lever under the action of thrust.

**Figure 8:**
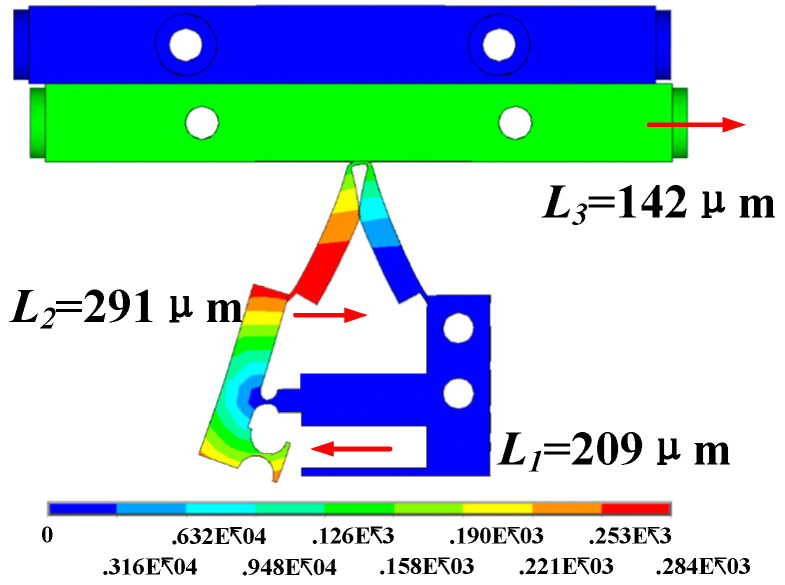
Lateral deformation of the piezoelectric actuator.

The deformation of the flexure hinge structure and horizontal displacement of the slider are shown in Figure 8. In the lever structure, when the horizontal displacement of dynamic application point is 209 μm, the horizontal displacement of the resistance application point is 291 μm. Through calculation, the amplification factor of the lever structure in the simulation is 1.4 times. The theoretical magnification is 2.1. The reduction of magnification is due to the absorption of part of the force due to the whole deformation of the lever. The bending deformation of the flexible hinge in the lever structure requires a certain amount of energy. The symmetrical deformation of both sides of the flexure hinge structure reflects its stable driving performance. The coupling displacement of the driving foot makes the slider move 142 μm horizontally.

**Figure 9:**
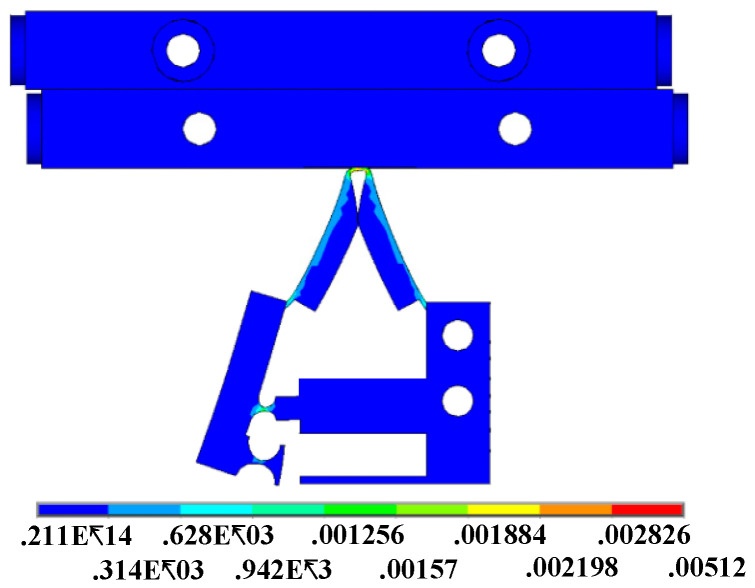
Equivalent stress distribution of the flexible hinge structure.

In the process of motion, the flexible hinge structure in the model will produce the stress to recover to the initial state due to the deformation. Figure 9 shows the distribution of the equivalent stress when the flexible hinge structure has the maximum deformation. The mechanical strain in the figure is concentrated on the hinge, driving foot, and outside of the triangular structure. The maximum mechanical strain occurs on the driving foot which is close, in touch, with the slider. According to Equation (5), the output of clamping force can be increased by changing the angle α between the structure of the triangular flexible hinge and the horizontal direction. However, for the sake of ensuring the normal operation of the driving foot. While increasing the angle to improve performance, it is also essential to take into account that the mechanical strain of the driving foot cannot exceed its bearing range.

## 4. Performance Analysis

**Figure 10:**
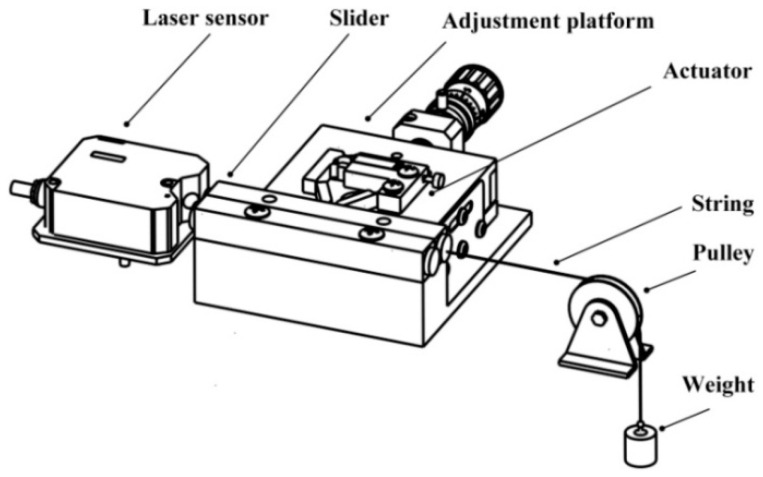
Mechanical load measurement system.

**Figure 11:**
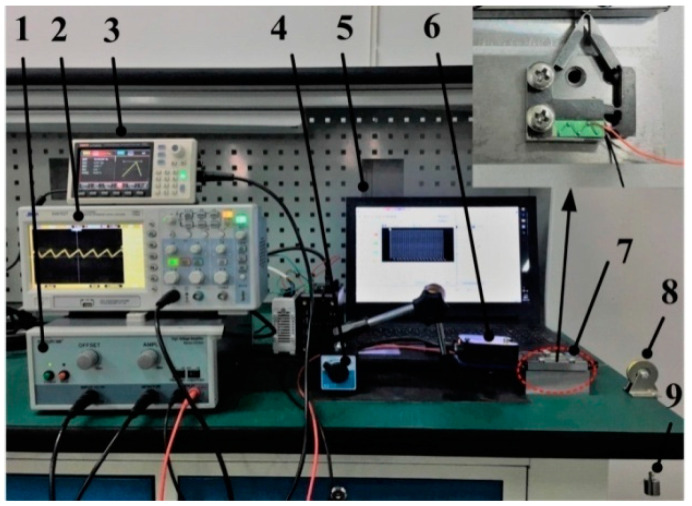
Experimental platform of the proposed stick-slip piezoelectric actuator (1. High Voltage Amplifier, 2. Oscilloscope, 3. Signal Generator, 4. Magnetic Base, 5. PC 6. Laser Sensor, 7. Prototype, 8. Pulley, 9. Weight).

According to the above analysis results, a 5 mm × 5 mm × 16 mm piezoelectric ceramic stack (NAC2013-H16, COREMORROW, Harbin, China) is added to the optimized flexure hinge structure. The hinge structure is welded by Al7075 and 65Mn. The maximum stroke of the cross-roller linear guide (VR4-80H×7Z, THK, Japan) is 58 mm. The assembled actuator and cross-roller linear guide are fixed on the corresponding platform by screws, and the pre-pressure between them is adjusted to 80 N. A string is fixed on the right side of the slider, and it is connected with a suspended weight through a fixed pulley. The pulley-string-weight is used as the load of the slider. The laser sensor (LK-H008, KEYENCE, Osaka, Japan) with a measuring range of ±0.5 mm detects it on the left side of the slider, which is used to detect and record the output performance of the slider, such as displacement or velocity. Figure 10 shows the mechanical load measurement system. In the experimental platform, the ramp voltage applied to the actuator is generated by the signal generator (UTG930, UNI-T, Dongguan, China), and the amplitude of the voltage is amplified by the high voltage amplifier (HA-820, PINTECH, Guangzhou, China). The oscilloscope is used to observe the stability of voltage waveform and adjust the voltage peak to peak with the high voltage amplifier. The magnetic base is used to adjust and fix the position of the laser sensor, and the data detected by the laser sensor will eventually be stored in the PC, as shown in Figure 11.

**Figure 12:**
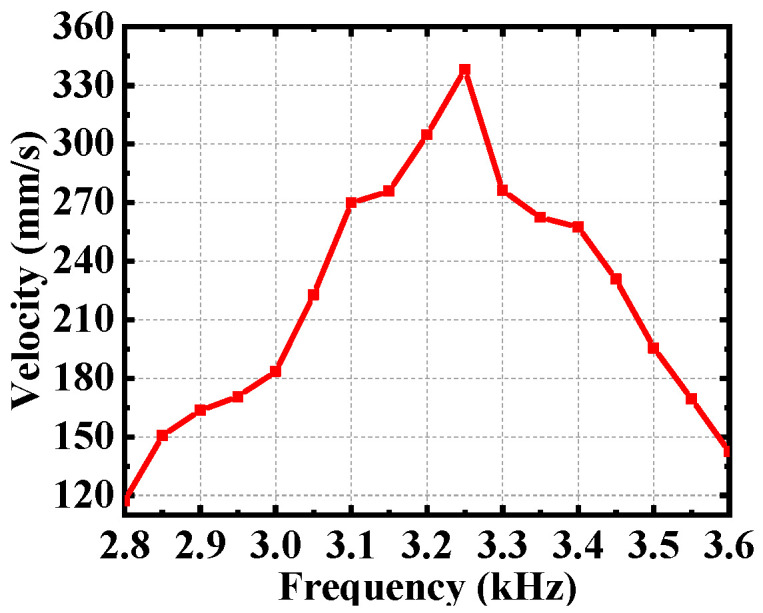
The relationship between drive frequency and the speed of the slider.

Firstly, the relationship between driving performance and excitation frequency is analyzed. Using ANSYS 15.0 (Ansys, Canonsburg, PA, USA) obtains the optimal resonance frequencies of 3.25 kHz in Modal analysis. Then, for the sake of facilitating comparison, the best excitation amplitude of 150 V is used. Finally, the parameter analysis of the frequency range is carried out to verify whether the frequency is the optimal excitation frequency, as shown in Figure 12. The slider speed increases with the rise of excitation frequency reaches the peak value at 3.25 kHz and then decreases rapidly. The result shows that 3.25 kHz is the best excitation frequency in this range.

**Figure 13:**
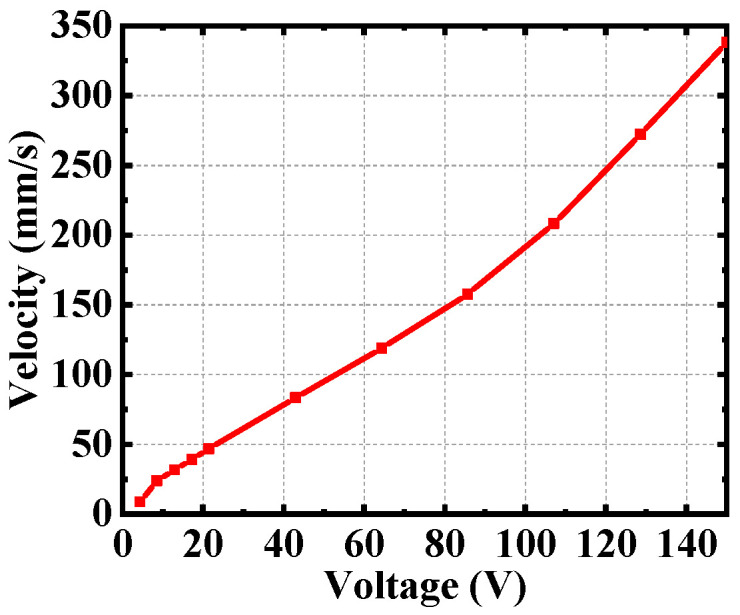
Relationship between drive voltage and the speed of the slider.

Through the above analysis, choose 3.25 kHz as the excitation frequency. The relationship between driving performance and excitation amplitude is studied, as shown in Figure 13. When the excitation amplitude is lower than 5 V, the slider cannot be driven effectively. When the excitation amplitude is greater than 5 V, the slider speed increases linearly as the excitation amplitude grows approximately. The optimum velocity is reached at the maximum excitation amplitude of 150 V.

**Figure 14:**
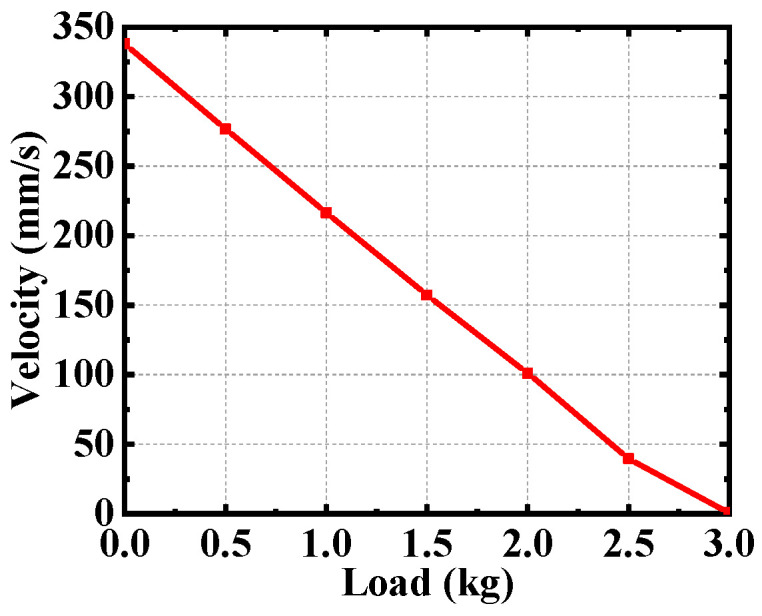
Load performance of the piezoelectric actuator.

When the excitation amplitude is 150 V and the excitation frequency is 3.25 kHz. Figure 14 shows the relationship between load capacity and velocity. With the increase of load, the sliding velocity decreases linearly. The velocity of the slider tends to zero when the load is 3 kg. Therefore, the maximum load that the piezoelectric actuator can bear is 3 kg.

**Figure 15:**
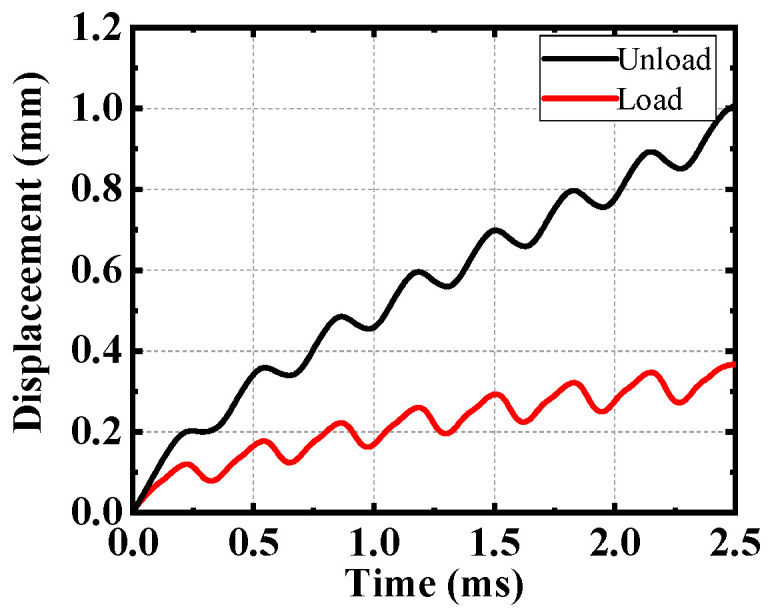
The relationship between displacement and time of the slider.

When the excitation frequency is 3.25 kHz and the excitation amplitude is 150 V, the sampling period of laser sensor is 20 μs and the average number of times is 1. As the voltage increases, the deformation of the flexure hinge structure leads to the increase of clamping force, which drives the slider to move laterally. Then, the driving voltage drops rapidly, the flexure hinge structure quickly recovers the deformation, the reverse friction force reacts on the slider. The rapid decrease of adhesion leads to less friction and short action time. At the same time, due to the inertia, the slider keeps a positive movement trend. To sum up, the slider can only produce a small reverse displacement. Therefore, the movement trajectory of the slider is the curve of the ascending step. After loading a 1.5 kg load, the slider has large reverse displacement in the process of motion, caused by the low velocity and reaction of loads, as shown in Figure 15. For a more clear and direct description, the performance results of proposed PEA are shown in Table 5. The displacement resolution varies with the applied voltage. In theory, the smaller the applied voltage within the driving voltage range, the higher the resolution of the PEA. However, the sensor itself has a resolution, and if the displacement is too small, it cannot be detected. After testing, the minimum resolution is 0.01 μm.

## 5. Conclusions

In order to adapt to different working requirements, flexible hinges are often added to the linear PEA to diversify the design of the whole structure. Among them, the combination with displacement amplification structure can improve the performance of the PEA. In the previous research of the PEA, a single displacement amplification structure was used for combination. On this basis, this paper combines two kinds of displacement amplification structures, constructed a two-stage amplification flexible hinge structure, and use it in the PEA to greatly improve the drive performance.

The structure adopted in the PEA has the characteristics of amplifying the output displacement and clamping force. In this paper, the amplification characteristics of the structure are analyzed theoretically. The structure was optimized by parametric analysis. Then, the theoretical calculation results were confirmed by FEA. The operation manifestation stated that under the excitation frequency of 3.25 kHz and the excitation amplitude of 150 V, the peak no-load output speed was 338 mm/s. The maximum load capacity was 3 kg. The structure improved the utilization ratio of piezoelectric ceramics. It solved the problems of unstable clamping force and insufficient load capacity. On the premise of ensuring the output performance, reducing the working frequency can improve the control accuracy. Further improvement of control accuracy is a problem to be studied.

## Figures and Tables

**Table 1:** Material parameters of flexible hinge structure.

Materials	Elasticity Modulus N/m^2^	Poisson’s Ratio	Density kg/m^3^
Constructional steel	2 × 10^11^	0.3	7850
Spring steel 65Mn	2.26 × 10^11^	0.3	7810
Alloy Al7075	7.17 × 10^10^	0.33	2810

**Table 2:** Parametric analysis of lever width.

Lever Width *L*_4_/mm	Contact Area Clamping Force *F_oy_*/N	Horizontal Displacement of Lever Side Surface *L*_1_/µm	Horizontal Displacement of Lever Top *L*_2_/µm	Horizontal Displacement of Slider *L*_3_/µm
3.5	1000	100	153	103
4.0	1008	120	162	104
4.5	1015	121	174	106
5.0	1003	105	163	103
5.5	999	92	160	101

**Table 3:** Parametric analysis of the distance between the hinge and the base.

Distance Between Hinge and Lever *L*_5_/mm	Contact Area Clamping Force *F*_oy_/N	Horizontal Displacement of Lever Side Surface *L*_1_/µm	Horizontal Displacement of Lever Top *L*_2_/µm	Horizontal Displacement of Slider *L*_3_/µm
2.0	1015	121	174	106
1.5	1045	137	190	109
1.0	1078	146	212	114
0.5	1110	160	232	117
0.0	1152	178	263	125

**Table 4:** Parametric analysis of hinge radius.

Hinge Radius *R*_1_/mm	Contact Area Vlamping Force *F*_oy_/N	Horizontal Displacement of Lever Side Surface *L*_1_/µm	Horizontal Displacement of Lever Top *L*_2_/µm	Horizontal Displacement of Slider *L*_3_/µm
1.20	1152	178	263	125
1.25	1170	180	273	129
1.30	1181	194	280	132
1.35	1194	201	287	139
1.40	1201	209	291	142

**Table 5:** Performance results of the proposed PEA.

Type of the PEA	Voltage (Vp-p)	Frequency (kHz)	Free-Load Velocity (mm/s)	Maximum Load (kg)
The PEA using a two-stage flexure hinge structure	150	3.25	338	3

## Data Availability

Data available on request from the authors.
